# Progressive myoclonus epilepsy in Gaucher Disease due to a new Gly–Gly mutation causing loss of an Exonic Splicing Enhancer

**DOI:** 10.1007/s00415-018-9084-4

**Published:** 2018-10-31

**Authors:** Rodolfo Tonin, Serena Catarzi, Anna Caciotti, Elena Procopio, Carla Marini, Renzo Guerrini, Amelia Morrone

**Affiliations:** 1 Clinic of Paediatric Neurology and Laboratories, A.O.U Meyer, Viale Pieraccini n.24, 50139 Florence, Italy; 2 Department of NEUROFARBA, University of Florence, Florence, Italy; 3 Metabolic Unit, A.O.U Meyer, Florence, Italy

**Keywords:** Gaucher Disease, *GBA* gene, Synonymous mutation, Exonic splicing enhancer, Exon skipping, Progressive myoclonic epilepsy

## Abstract

**Background:**

Patients with Gaucher Disease (GD) exhibit three phenotypes, including type 1 (non-neuronopathic), type 2 (acute neuronopathic), and type 3 (subacute neuronopathic).

**Aim:**

Identifying which *GBA* changes represent benign polymorphisms and which may result in disease-causing mutations is essential for diagnosis and genotype/phenotype correlations but is often challenging.

**Results:**

Here, we describe a patient with type 3 GD, presenting with drug-resistant epilepsy, who bears a set of *GBA* polymorphic variants including the novel c.363A > G (Gly82Gly) synonymous mutation. *In silico* predictions, mRNA and functional studies revealed that the new Gly82Gly mutation causes skipping of *GBA* exon 4, leading to a severe reduction of the wild type *GBA* mRNA. This is the first report of a synonymous change causing GD through loss of an exonic splicing enhancer sequence.

The synonymous mutation is in trans with the Asn188Ser missense mutation, thus making the Asn188Ser responsible for the patient’s phenotype and strengthening the association of Asn188Ser with the particular neurological phenotype of type 3 GD.

**Conclusion:**

We strengthen the association of Asn188Ser with the type 3 GD phenotype and progressive myoclonus epilepsy. Our data confirm that *in silico* predictions and mRNA analysis are mandatory in discriminating pathological mutations from the background of harmless polymorphisms, especially synonymous changes.

## Introduction

Gaucher disease (GD) is an autosomal recessively inherited metabolic defect due to deficiency in the lysosomal enzyme β-glucosidase (EC 3.2.1.45, also referenced as glucosylceramidase or β-glucocerebrosidase) causing the lysosomal accumulation of glucosylceramide. GD is the most common lysosomal storage disease with a prevalence ranging from 1/100,000 to 1/855 in Ashkenazi Jews [26].

GD patients exhibit a broad spectrum of manifestations including hepatosplenomegaly, anemia, thrombocytopenia, bone disease and neurological symptoms. Based on the presence and progression of neurological symptoms, GD is classically divided into type 1 (nonneuronopathic), type 2 (acute neuronopathic), and type 3 (subacute neuronopathic) forms [1, 18, 37], although this distinction does not always correspond to sharply distinct phenotypes [15].

GD type 1 affects the majority of patients (95% in Europe and USA, but less in other regions) with onset in childhood or adulthood. GD type 2 is the more severe form of the disease with early post-natal onset and survival of up to 2 years of age while GD type 3, has infantile or juvenile onset, and usually allows survival into past early adulthood [32].

Recently, a clinical association has been reported between the presence of mutations in the β-glucosidase gene and Parkinsonism [17, 34].

The gene encoding β-glucosidase (*GBA*), located in 1q21, comprises 11 exons and 10 introns spanning 7.6 kb. A highly homologous pseudogene (*GBAP*) is located 16 kb downstream of the *GBA* gene, with a 96% match in sequence identity and the same organization, thus complicating mutation detection strategies [14, 19, 20, 35].

To date over 470 mutations have been described in the *GBA* gene, including 362 missense/nonsense mutations, 25 splicing mutations, 35 small deletions, 15 small insertion and 21 complex rearrangements (HGMD professional database; http://www.biobase-international.com/product/hgmd).

The most frequent *GBA* mutations are the c.1226A > G (Asn370Ser), which correlates with non-neuronopathic GD type 1, and the c.1448T > C (Leu444Pro), which prevalently correlates with the neuronopathic forms of the disease [32].

Correctly identifying disease-causing mutations from the background of harmless nucleotide polymorphisms/substitutions is crucial when investigating human genetic diseases. Here, we describe the biochemical and molecular characterisation of a 17 years old patient with type 3 GD, apparently bearing only one clear-cut mutation in the *GBA* gene. We provide evidence that a new synonymous change resulted in the second disease causing allele in this patient’s *GBA* gene.

## Patient and methods

### Case report

The patient, a 17-year-old girl, born from healthy consanguineous Italian parents, was delivered at full term. Pregnancy was uneventful and psychomotor development was normal.

**Fig. 1:**
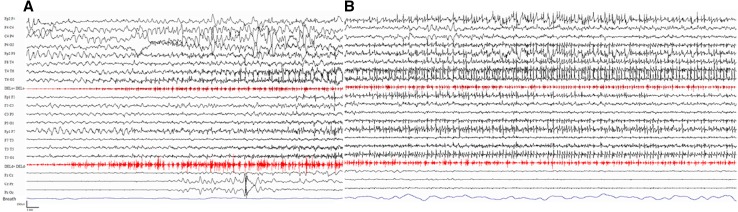
Polygraphic EEG recording. A nocturnal seizure with a crescendo of myoclonic/clonic jerking and evolving into a tonic-clonic seizure is showed. **a** The initial part of the seizure highlights the onset as single, repetitive and rhythmic myoclonias. **b** The final part demonstrates the progressive recruitment of the hyperexcitable neurons and the evolution into a tonic and fast clonic jerking phase. All seizure lasted several minutes, EEG amplitude recording was 250 microV, speed: 20 mm/second. DELs = left deltoid muscle; DELd = right deltoid muscle

At age 11 years a first sleep-related tonic-clonic seizure, lasting several minutes appeared. A first EEG recording showed discharges of generalized spikes and polyspike-waves together with multifocal, centro-parieto-temporal paroxysmal activity. Brain MRI was unrevealing. Treated with valproic acid and clobazam, she was seizure-free for nearly 2 years. At age 13-years, seizures relapsed and over time became drug-resistant despite different antiepileptic drug combinations, including ethosuximide, lamotrigine, benzodiazepines, acetazolamide, levetiracetam, topiramate, lacosamide and barbiturates. Seizures occurred 2–3 times per month, predominantly during sleep, as tonic-clonic, lasting several minutes and occasionally requiring acute treatment with rectal diazepam. In the same period, parents also noticed daily episodes of loss of contact and interruption of motor activity with a slight head drop and eyelid fluttering, lasting 10–20 s. Long-term video-EEG monitoring captured sleep-related seizures, with the tonic-clonic phase being preceded by a crescendo of myoclonic and clonic jerks (Fig. 1). We also recorded several episodes of ictal eyelid myoclonia with absences associated with polyspike and wave discharges. The interictal EEG was severely abnormal with frequent discharges of generalized or multifocal paroxysmal activity, yet the most interesting features were observed during sleep with activation of severe paroxysmal discharges and absence of a recognizable physiological EEG pattern. EEG also showed a prominent photosensitivity. During intermittent photic stimulation, we recorded a generalized photoparoxysmal response often provoking eyelid myoclonia.

After the onset of seizure, she also manifested cognitive regression, leading to mild-moderate cognitive impairment. Haemoglobin levels and white blood cell values were normal, although at the lower end of normal limits. Trombocytopenia was not found. At age 16 years, neuropsychological testing showed a global IQ score of 65 (WISC-IV). Neurological evaluation revealed mild dysarthria, increased deep reflexes, mild dysmetria, inability to walk on toes, and upper extremity tremor and myoclonus. Somatosensory and visual evoked potentials revealed responses of abnormally high amplitude (grossly enlarged potentials).

Clinical features, particularly their onset and evolution, EEG features, and evoked potentials were suggestive of progressive myoclonus epilepsy. Abdominal ultrasound and MRI revealed also a mildly enlarged liver. The magnetic resonance (MR) imaging bone marrow burden (BMB) score was 3. Thus, considering all the above findings combined with age at symptoms onset, we investigated the patient for Lafora disease and lysosomal disorders, including GD.

At clinical evaluation spleen and liver were of normal size. At abdominal MRI the liver was at the upper limit of normal (2626 cc). There was neither anemia nor thrombocytopenia. Since, in keeping with GD, β-glucosidase activity in blood spots was deficient, we performed *GBA* gene screening and skin biopsy for further molecular and enzyme activity assays to confirm the diagnosis.

Once the diagnosis was confirmed the enzyme replacement therapy (ERT) was instituted. At present, the patient, aged 17 years, exhibits drug-resistant epilepsy with daily myoclonic and monthly tonic-clonic seizures, often while asleep and during her periods, prominent photosensitivity, mild-moderate cognitive impairment with dysarthria and upper limb tremor and an abnormal bone marrow burden. Haematological values and BMB were unchanged. The clinical picture is fluctuating, yet slowly progressive, and ERT is of no benefit on neurological symptoms, considering also this treatment does not pass the brain blood barrier.

### Enzymatic assays

Human skin fibroblasts from the patient were cultured in Dulbecco’s modified Eagles with foetal bovine serum (10%) and antibiotics. β-glucosidase enzyme activity was measured on leukocytes and fibroblasts using the fluorogenic substrate 4-methylumbelliferyl-β-glucopyranoside in the presence of sodium taurocholate and Triton X100 [4, 5, 30].

### Mutation nomenclature

Mutations are described as recommended, considering nucleotide + 1 the A of the first ATG translation initiation codon [6, 7] (http://www.hgvs.org/mutnomen/). Nucleotide numbers are derived from the *GBA* cDNA (GenBank reference sequence NM_000157.1).

β-glucosidase protein mutation nomenclature does not start with the first ATG codon unless required by standard nomenclature due to a processed leader sequence (reference sequence AAC63056.1). The current nomenclature envisages the mutations to be named without “p.”.

### Mutational analysis of the *GBA* gene

Genomic DNA from the patient and her parents was isolated from peripheral blood lymphocytes using the QIAsymphony instrument as recommended by the manufacturer (Qiagen, Hilden, Germany). The entire coding region and most intronic sequences of the *GBA* gene were amplified in three fragments using primers that selectively amplify the gene and not the pseudogene [23], purified PCR products were directly sequenced on ABI PRISM 3130 XL Genetic Analyzer using Big Dye Terminator chemicals (Applied Biosystems, Foster City, USA).

### Screening of new variants and in silico analysis

The presence and frequency of the newly identified variants were initially checked in dbSNP database (http://www.ncbi.nlm.nih.gov/SNP/). They were also examined in the 1000 Genomes project database (http://browser.1000genomes.org/index.html), in the Exome Sequencing Project (ESP) database which collects over 6500 alleles (http://evs.gs.washington.edu/EVS/) and in the Exome Aggregation Consortium (ExAC), which provides a data set spanning over 120,000 alleles (http://exac.broadinstitute.org).

To evaluate the effect of the new mutation on the splicing of the GBA gene we used the Alamut software (http://www.interactive-biosoftware.com), a mutation interpretation software integrating the results of 5 different algorithms (SpliceSiteFinder, MaxEntScan, NNSPLICE, GeneSplicer, Human Splicing Finder).

The repercussion of the new variant on the Exonic Splicing Enhancers (ESEs) and Exonic Splicing Silencers (ESSs) was also evaluated with Rescue ESE (http://genes.mit.edu/burgelab/rescue-ese/) and ESE Finder (http://exon.cshl.edu/ESE/).

### RNA isolation and retrotranscription

Total RNA was isolated from whole blood samples using the NucleoSpin RNA Blood kit (Macherey–Nagel, Düren, Germany). RNA integrity and its concentration were checked by 1% agarose gel and Nanodrop® ND-1000 Spectrophotometer (Nanodrop Technologies, Wilmington, USA).

RNA reverse transcription was carried out as follows:

1. 160 ng of total RNAs were reverse transcribed using both random hexamers and a specific *GBA* 3′ UTR primer (5′ GGCCTCCAGCCCCTG 3′) by using Superscript II First Strand kit (Invitrogen, Carlsbad, USA) according to the manufacturer’s instructions.
2. 200 ng of total RNAs were reverse transcribed in 40 µl of final volume in a reaction mixture containing 4 µl TaqMan RT buffer 1×, 5.5 mM MgCl_q_, 500 µM each dNTP, 2.5 µM random hexamers/specific *GBA* 3′ UTR primer, 0.4 U/µl RNase Inhibitor and 1.25 U/µl Multi-Scribe Reverse transcriptase provided by Applied Biosystems. The profile of the one-step reverse transcriptase was: 10 min at 25 °C, 30 min at 48 °C and 2 min at 95 °C.

The reverse transcripts obtained from the second method were used for quantitative real-time analysis.

### Retro transcribed PCR (RT-PCR) analysis on lymphocytes samples

RT-PCR analysis was performed using 2 µl of retro-transcribed products from method 1 as template and the used primers were *GBA* exonic 2F 5′ GAATGTCCCAAGCCTTTGAGTA 3′ and *GBA* exonic 10R, 5′ CGACCACAACAGCAGAGC 3′. PCR conditions are the following: 4 min at 95 °C, then submitted to 33 cycles of amplification at 95 °C for 30 s, 58 °C for 1 min, and 72 °C for 3 min, final extension cycle at 72 °C for 7 min. Normalized Volume quantification obtained with ChemiDoc MP imager (Bio-Rad, Hercules, USA) [38].

### Quantification of *GBA* mRNA on lymphocytes samples

The quantification of the here evidenced alternative forms of *GBA* mRNAs was performed using a quantitative realtime RT-PCR method, based on TaqMan™ technology. Probes and primers were selected by the “Primer Express” software (Applied Biosystems).

To detect the wild type *GBA* mRNA, the following probe and primers were chosen: probe 5′ ACAGGCCTGCTACTGACCCTGCAGC 3′ labelled with FAM, located on exon 3/4 junction, forward primer (F1): 5′ GCCCATCCAGGCTAATCACA 3′ which hybridizes on *GBA* exon 3, reverse primer (R1): 5′ CCTCCAAATCCCTTCACTTTCTG 3′ located on *GBA* exon 4.

For the detection of the mutated *GBA* mRNA the following probe and primers were chosen: probe: 5′ ATATAACATCATCCGGGTACCCATGGCC 3′ labelled with JOE, located on *GBA* exon 5, forward primer (F2): 5′ ACACGGGCACAGGAATCG 3′ which hybridizes on exon 3/5 junction, reverse primer (R2): 5′ GTGCGGATGGAGAAGTCACA 3′ located on *GBA* exon 5.

The variations in input amounts of control and patients’ RNA samples were relatively quantified by the use of 18S control assay (Applied Biosystems), applying the ΔΔCt method as previously reported [24]. PCR analysis was performed using 25 ng of cDNA in a reaction mixture containing 300 nM of forward and reverse primers and 200 nM of the fluorescent probe, and 12.5 µl Universal masterMix. Plates were treated for 2 min at 50 °C, 10 min at 95 °C and then submitted to 40 cycles of amplification at 95 °C for 15 s, 60 °C for 1 min in the ABI Prism 7000 Sequence Detector PE Applied Biosystems.

### Minigene functional studies

**Fig. 2:**
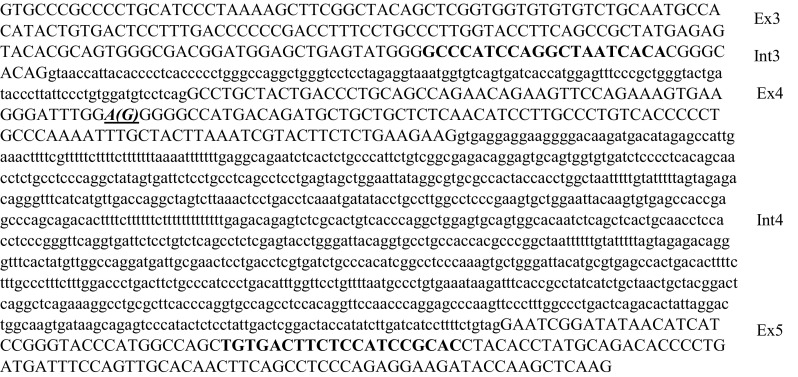
Sequences of the fragments inserted into minigenes. Capital letters indicate exons. Bolded sequences indicate Primer F1 and R2, used in the RT-PCR analysis. The bolded, italic and underlined base indicates the c.363A > G mutation

Minigene expression systems [8] were employed to further characterise the effect of the c.363A > G (Gly82Gly) on GBA mRNA. Minigenes carrying wild type and mutated portions of GBA (exons 3–5 and intron 3 and 4) were synthesised and inserted into pCDNA3.1 plasmid by Biomatik (Biomatik Corporation, Cambridge, Canada), clone ID: M2370-1 and M2297-1 (Fig. 2). Plasmid carrying wild type and mutated minigenes were transformed into E-coli SoloPack Gold Competent Cells (Agilent Technologies, Santa Clara, USA) following the manufacturer’s instructions. We obtained a great quantity of each recombinant vector extracted using the Endofree plasmid extraction kit (Macherey Nagel, Düren, Germany). The integrity of the DNA and the fidelity of the inserted sequences were verified by sequencing both strands. Normal and mutant vectors were transiently over expressed in COS-1 as described previously [11]. Total RNA was extracted and reverse transcribed after 48 h transfection. Total mRNA extraction and cDNA synthesis are described above (see method 1 for mRNA synthesis).

The used oligonucleotides are above described (F1 and R2); RT PCR conditions are also above described for the RT-PCR analysis performed on fibroblasts samples.

### Western blotting

β-glucosidase western blot was performed in fibroblasts of patient and controls. Cells were lysed in ice-cold RIPA buffer (50 mM Tris/HCl pH 7.5, 1% Triton X-100, 150 mM NaCl, 100 mM NaF, 2 mM EGTA and protease inhibitor cocktail P8340 Sigma Aldrich, Saint Louis, USA) and after 15 min on ice were centrifuged at 11,600*g* for 10 min. Protein concentrations were determined on the supernatants by the BCA method (Sigma Aldrich). Equal amounts of total proteins (50 µg) were loaded in each line and were subjected to 12% SDS–PAGE gel and electro transferred to nitrocellulose membrane (GE Healthcare, Milan, Italy) that was probed with anti-GBA monoclonal antibodies (Ab-CAM, ab55080; Sigma Aldrich WH0002629M1), anti-GBA polyclonal antibody (Sigma Aldrich HPA006667) and with anti-b-actin polyclonal antibodies (Sigma Aldrich) to normalize. Secondary antibodies conjugated to alkaline phosphatase (Sigma Aldrich, Saint Louis, USA) were used to detect antigen–antibody complexes, revealed by AP Conjugate Substrate kit (Bio-Rad). Bands corresponding to GBA proteins were quantified by ChemiDoc and Image Lab software (Bio-Rad) [38]. The same filters were also probed with anti-β-actin antibodies for cell lysates normalization.

### Statistical analysis

Statistical analysis was carried out using Image Lab software (Bio-Rad) and Microsoft Excel 97 SR-2. P values less than 0.05 were considered statistically significant.

## Results

### Biochemical analysis and *GBA* gene mutational analysis

Activity of β-glucosidase performed on leukocytes extracts was 1.9 nmol/mg/h (control range 4.8–14 nmol/mg/h). The partial lack of β-glucosidase residual activity is in keeping with the patient’s phenotype.

Routine sequencing analysis of the *GBA* gene identified only one clear-cut mutation: the c.680A > G; (Asn188Ser) (p.Asn227Ser if considering the processed leader sequence) [22], together with nine apparently polymorphic nucleotide variants. Of these, only the c.363A > G (Gly82Gly) (or p.Gly121Gly if considering the processed leader sequence) had not been previously reported in the dbSNP-1000 genomes and ExAC servers (http://www.ncbi.nlm.nih.gov/SNP/; http://browser.1000genomes.org/index.html; http://exac.broadinstitute.org). The patient’s father is carrying the c.363A > G (Gly82Gly) variant, inherited from the paternal line of his family, while the c.363A > G (Gly82Gly) variant was inherited from the patient’s mother. The mother’s parents died before genetic analyses could be extended to family members.

The possible effect of the c.363A > G substitution on the *GBA* mRNA splicing process was *in silico* investigated and no significant changes were predicted to affect 5′ and 3′ splice site signal strengths. The repercussion of the new variant on the Exonic Splicing Enhancers (ESEs) and Exonic Splicing Silencers (ESSs) was evaluated with RescueESE (http://genes.mit.edu/burgelab/rescue-ese/) and ESE Finder (http://exon.cshl.edu/ESE/). The c.363A > G substitution was indicated to cause the loss of an ESE within *GBA* exon 4.

### RT-PCR analyses on lymphocyte samples

**Fig. 3:**
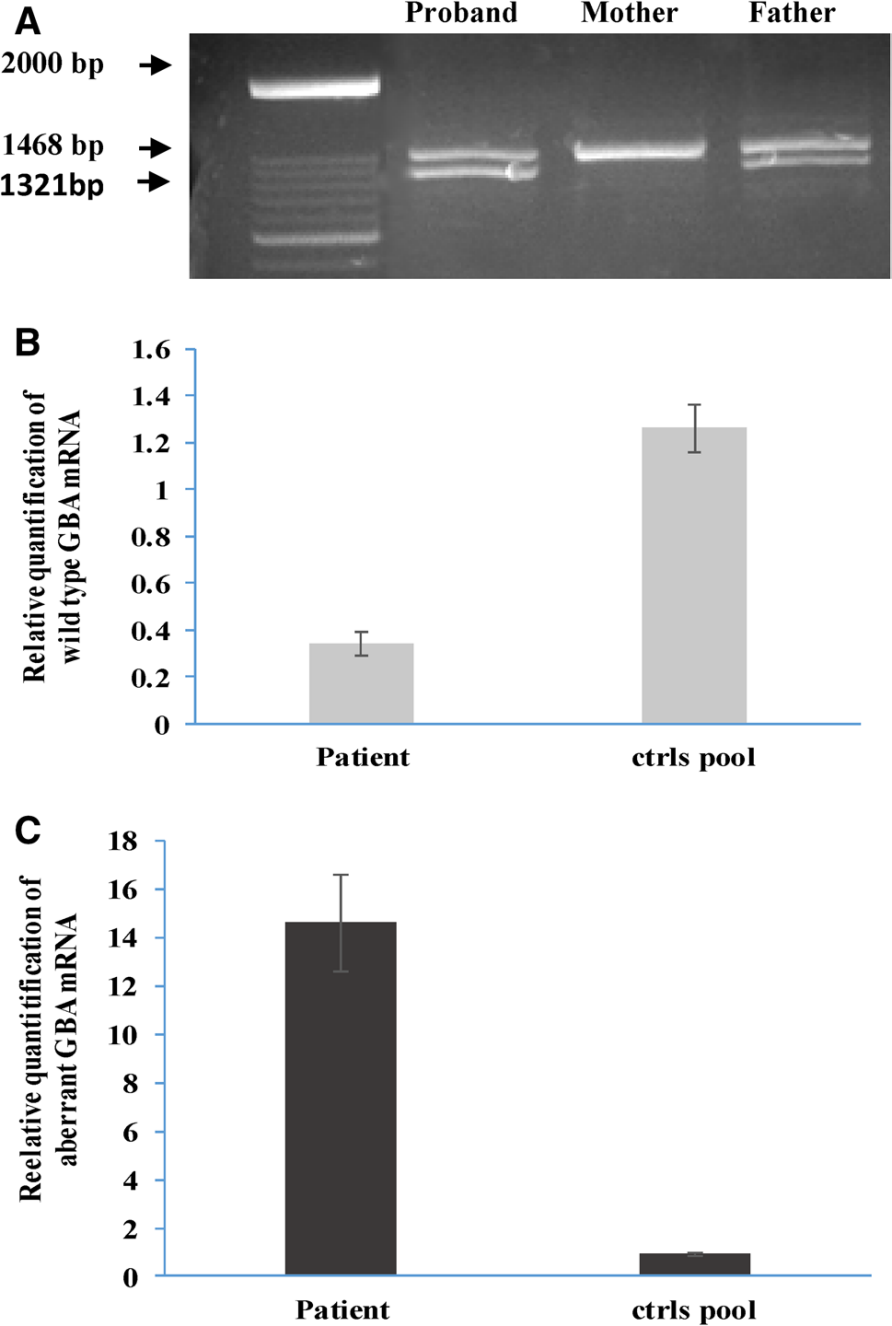
Retro-transcribed and Real-time PCR analyses on cDNA obtained from lymphocyte samples. **a** RT-PCR analysis of patient, and her parents. Normal size band is 1468 bp, while the mutant band missing exon 4 is 1321 bp in length. **b, c** Real-time quantification of: **b** wt *GBA* mRNA; **c** aberrant *GBA* mRNA. 18S mRNA quantity was used to normalize data

The possible loss of an ESE and its impact on the *GBA* mRNA splicing process was investigated by mRNA studies on proband, father and mother. RT-PCRs showed a unique amplified band in control samples and two transcripts, in the same proportion, in the proband and her father (Fig. 3A). Sequencing analysis revealed that the upper band of the father, the only band present in the control *GB*A mRNAs, was the correctly, wild type processed transcript containing all the expected *GBA* exons, while the lower band corresponded to an aberrant transcript lacking 147 bp, completely missing the *GBA* exon 4. At a protein level, the skipping of exon 4 causes a deletion of 49 amino acids (p. 103_151), that is supposed to give an “in frame” shorter protein. As expected, sequencing analysis of the patient’s upper band revealed the Asn188Ser mutation, given that the other allele generated the above described mutant mRNA transcript. Sequencing analysis of the father’s upper band revealed the wild type allele while the lower band was the aberrant transcript lacking exon 4.

### Real-time PCR analysis on lymphocyte samples

The quantities of wild type and mutant *GBA* mRNAs were evaluated by Real Time PCR analysis (Fig. 3 b, c). Quantitative analysis of the *GBA* gene mRNAs was performed by relative real-time PCRs, using probe and primers encompassing exon 3–4 junction (wt probe) and exon 3–5 junction (mutant probe). mRNAs were evaluated on leukocyte lysates of our trios and a pool of 10 healthy controls.

The wild type *GBA* mRNA, measured in the patient’s sample, was about one-third that of control values, confirming data from conventional RT-PCRs. The mutant *GBA* mRNA was present at a very high level in the patient’s samples i.e. a 14.5fold increase compared to controls. A small amount of aberrant mRNA was found in the control samples, thus implying that a low amount of aberrant splicing is naturally present.

### RT-PCR analyses on minigene systems

**Fig. 4:**
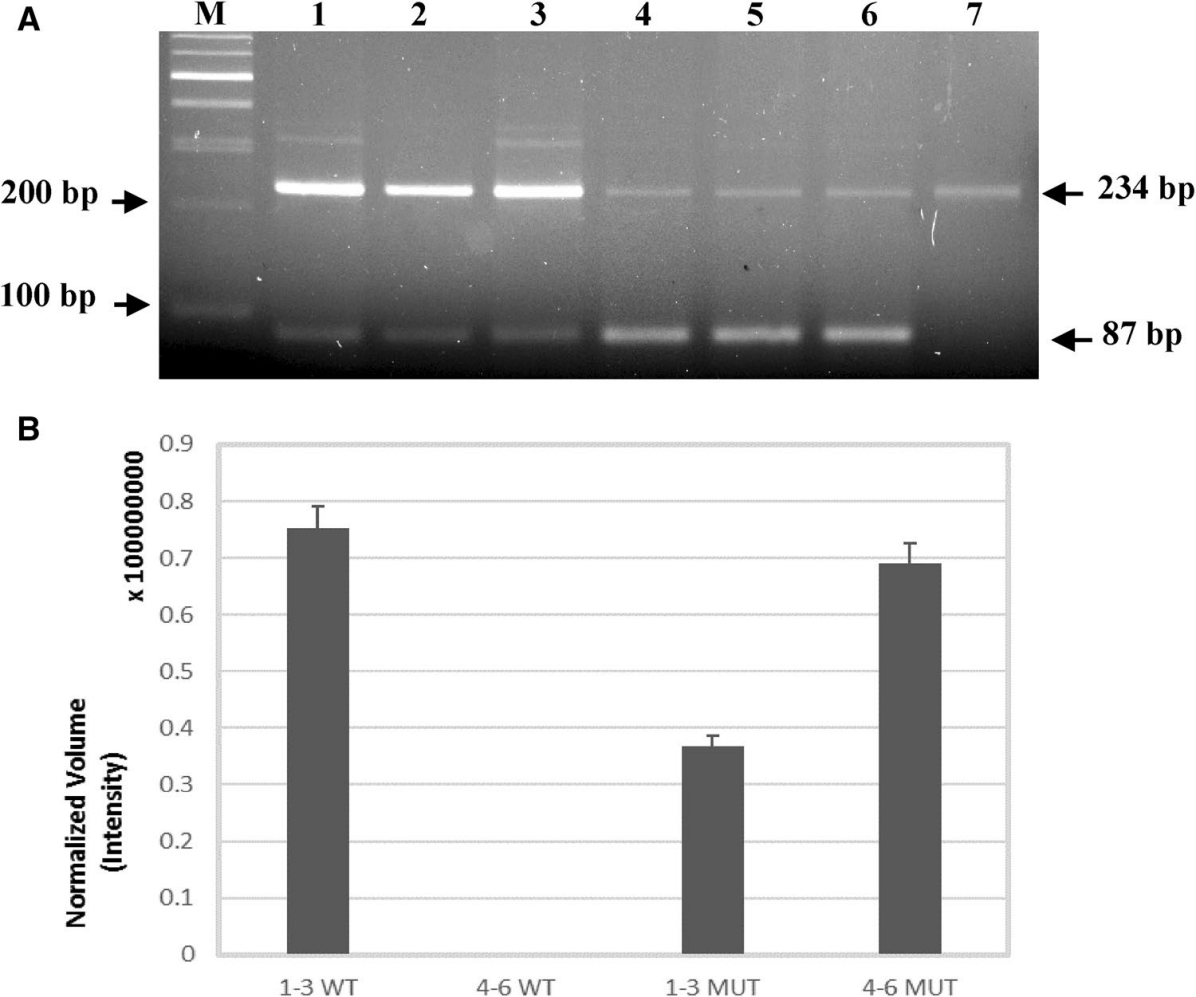
Minigene analysis. **a** RT-PCR analysis of COS1 cells transfected by systems reporting: *1–3*. WT minigenes, *4–6*. mutated minigenes; *7*. Amplified fragments of non-transfected COS-1; *M*. Molecular weight marker. **234 bp**. Amplified fragments containing *GBA* exon 4; **87 bp**. amplified fragments without *GBA* exon 4. Both fragments (243 bp and 87 bp) were sequenced on both strand. **b** Normalized Volume quantification obtained with ChemiDocMP imager (Bio-Rad). *1–3* wt minigenes; *4–6*. mutated minigenes; *WT* wt *GBA* mRNA; *MUT* Mutated *GBA* mRNA. Standard deviation is provided

Minigene expression systems were evaluated by RT-PCR analyses as previously described [8]. RT-PCR analyses confirmed an unbalanced expression of *GBA* transcripts (wild type and mutated) in the minigene systems (Fig. 4A). A background amplification value corresponding to the wild type GBA amplified band was detected in the non-transfected COS1 cells. Normalisation of the wild type bands to the value of non-transfected COS1 cells and the following quantification of bands volumes confirm that the c.363A > G mutation unbalances the expression of the *GBA* mRNAs causing an increment in the production of the *GBA* alternatively spliced mRNA (Fig. 4a, b). The wild type *GBA* mRNA, measured in the system transfected with wild type minigenes, was undetectable in the system expressing the mutated minigene (Fig. 4b). Conversely, levels of mutant *GBA* mRNA increased about twofold in the systems expressing the mutated minigenes as compared to the systems expressing the wild types (Fig. 4b).

### Western blot analysis

**Fig. 5:**
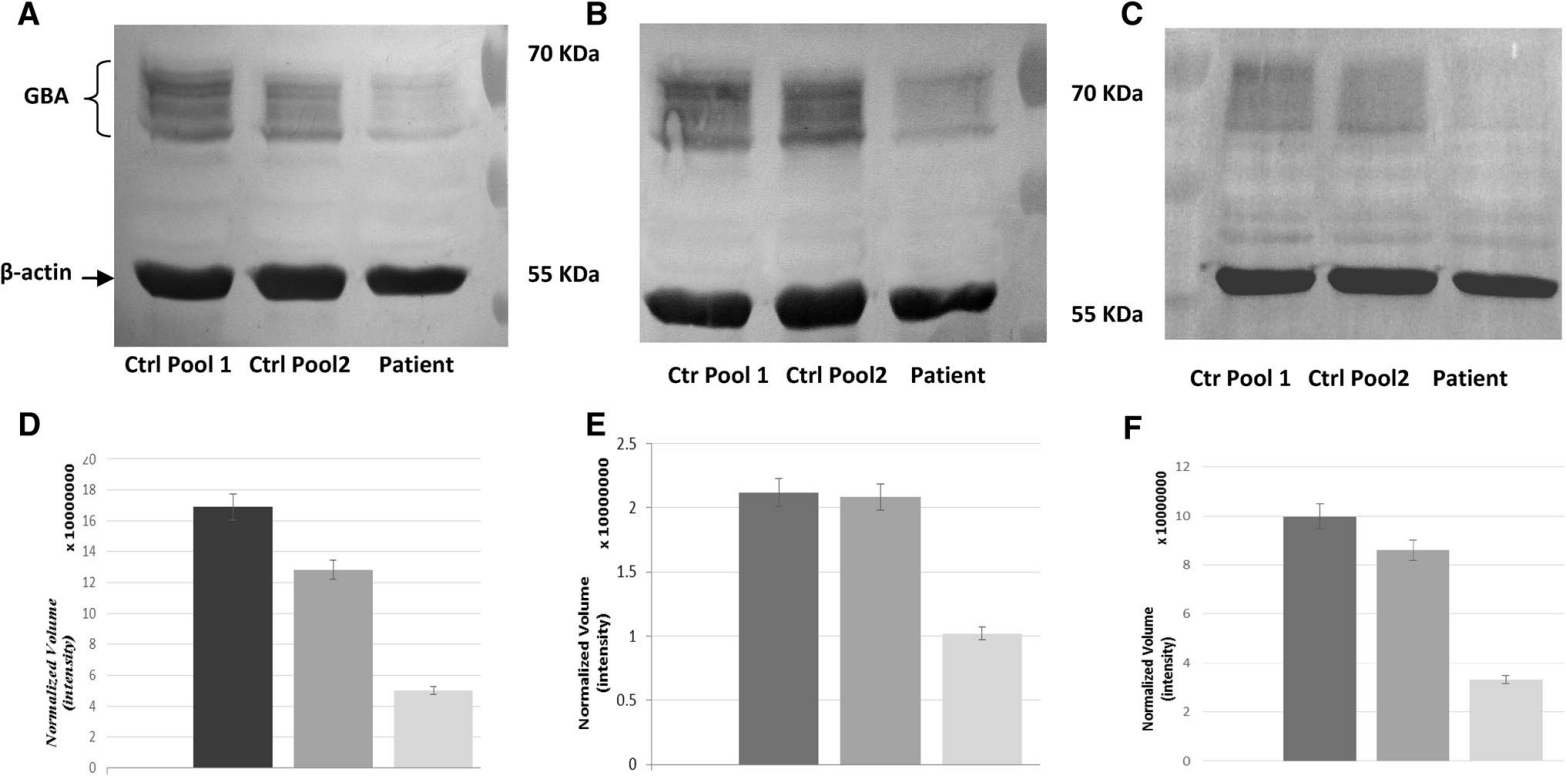
Western blots on fibroblast lysates probed with different anti human β-glucocerebrosidase antibodies. Western blots on fibroblasts lysates immunised against: **a** ab-CAM monoclonal anti-β-glucosidase (ab55080), **b, c** Sigma–Aldrich monoclonal anti-β-glucosidase (WH0002629M1 and HPA006667). **D–f** Normalized Volume quantification with ChemiDocMP imager (Bio-Rad), standard deviation is provided

Western blot analysis using anti-GBA monoclonal antibody showed a markedly reduced protein level in the patient’s fibroblasts compared with controls and to a normal amount of control protein (β-actin) (Fig. 5a). A multiband pattern was detected using either a second different monoclonal and one polyclonal anti-human acid β-glucosidase (Fig. 5b, c), this pattern corresponds to the differently glycosylated forms of the enzyme [27]. The strong reduction of the patient β-glucosidase protein quantity was confirmed with all the three different antibodies used. Quantization of band intensity is also reported (Fig. 5d–f).

## Discussion

Here, we describe the new c.363A > G Gly82Gly synonymous mutation, that we found in a patient with type 3 GD. Synonymous mutations do not alter the protein sequence and were previously considered as “silent” mutations i.e. without deleterious consequences on protein functions. Thus, their effects may have been underestimated and have probably been under-reported in the past. Synonymous mutations are now widely acknowledged to be able of causing changes in protein expression, conformation and function. Synonymous mutations have been associated with a constantly growing list of human diseases, now including over 50 entries [3, 21, 33]. Amongst metabolic diseases we recently identified silent mutations leading to Barth disease [12] and short-chain acyl-CoA dehydrogenase deficiency (SCADD) [39].

To the best of our knowledge, we here report the second silent mutation, which causes a splicing defect in the *GBA* mRNA processing leading to GD.

In the HGMD mutation database only 3 exonic mutations leading to GD are reported as disrupting the proper mRNA splicing process. Two of them are missense mutations, which alter the natural splice donor sites [10, 28], and one is a silent mutation introducing a cryptic donor splice site [8]. Therefore, we report the first silent mutation altering the proper splicing process through loss of an ESE sequence, hence resulting in GD. The correct recognition of the splice sites is strictly controlled and is dependent on intrinsic regulatory sequences. Among these, ESE sequences, which are located at varying distances from the splice sites, bind serine/arginine-rich splicing factors (SRSFs) that enhance the recognition of the splice sites [3]. Mutations within the ESEs exert an inhibitory effect on the binding of these proteins, resulting in a failure of the correct splicing and generating mutant mRNAs. The c.363A > G mutation generates a long stretch of G’s, which could, in fact, be regarded as the generation of two GGG triplets (spaced by a G). Multiple triplet Gs in exons are known to inhibit splicing by recruiting splicing inhibitory proteins from the hnRNPF/H family [16], so this could be the possible mechanism involved in the missplicing of the patient’s *GBA* mRNA.

The pathogenetic role of synonymous mutations is often difficult to identify and time consuming to confirm, so their effects have often been overlooked. This may be why the number of disease causing synonymous mutations has been underestimated in the past.

The loss of an ESE, due to the new *GBA* gene variant c.363A > G, resulted in the skipping of exon 4 in the mature *GBA* mRNA. This change leads to the in frame deletion of 147 nucleotides (c.308_454) in the mature mRNA and to the loss of 49 amino acids (Gly64_Glu112) in the GBA enzyme protein. Most of the lost amino acids are located in protein Domain III (residues 76–381 and 416–430), a (β/α)_8_ TIM barrel which contains the catalytic site [9]. We can expect that the short mutant protein lacks enzymatic activity, hence the new c.363A > G substitution can be considered as a null mutation, and we can assume that the patient’s phenotype is determined by the known c.680A > G Asn188Ser mutation. To better understand the effects of the Gly82Gly silent mutation we performed SDS page and western blot analysis on fibroblasts from patient and controls. These functional studies revealed that the patient had a significantly lower level of β-glucosidase protein compared to controls. These results allowed us to hypothesize that the shorter protein is probably unable to fold properly and to pass the quality control of the machinery of the endoplasmic reticulum, then being held in the reticulum and degraded, as reported [31].

Patients with type 3 GD exhibit different clinical manifestations but in recent years a subset of patients who developed a treatment refractory form of progressive myoclonus epilepsy has been identified. These patients shared several alleles including Asn188Ser [29].

The Asn188Ser mutation was first identified in Korean and Chinese patients, one of whom was homozygous for the Asn188Ser [36]. At the time of that report, none of the patients exhibited neurological signs, so they were all diagnosed as having type 1 GD [22].

This mutation was also identified by Park et al. 2003 in 4 GD type 3 patients who share progressive myoclonus epilepsy. In one of them, the Asn188Ser change was associated with the early frameshift mutation c.84-85insG, resulting in no protein product. In the other three patients it was recovered in combination with mutant alleles yielding proteins with various degrees of dysfunction. In all four patients, myoclonic seizures occurred from 12 years of age, suggesting a possible modifying role of the abnormal GBA enzyme on other proteins involved in epileptogenesis [29].

In 2004, a patient bearing the Asn188Ser in association with the severe Ser107Leu mutation was identified. The latter variant typically causes type 2 GD but the patient exhibited general seizures at the age of 11, visual seizures and myoclonus with moderate hepatomegaly, while classical clinical features such as anemia, thrombocitopenya, and bone pains were absent. Thus, also in this patient myoclonic epilepsy was attributed to the rare Asn188Ser variant [13]. In 2011 a couple of monozygous twins carrying the Asn188Ser / Asn188Ser genotype were reported [2]. One patient had severe visceral involvement, epilepsy and a cerebellar syndrome. The other twin, despite the same biochemical and molecular picture, did not manifest any symptom of GD. This latter work emphasizes the possible limitations in genotype-phenotype correlations, confirming the role of modifiers and/or environmental factors on the initiation and progression of GD; on the other hand, it introduces an additional case of myoclonic epilepsy caused by the Asn188Ser mutation [2].

Finally in 2014, Asn188Ser was found *in cis* with Gly265Arg in a GD type 3 patient in whom the second allele remained undetermined. Functional analysis on N188S transiently expressed in HEK cells showed 25% of residual GBA activity while it was nearly absent in the Gly265Arg expression system. Nonetheless, the cumulative effect of the two mutations was extremely deleterious; GBA activity of the corresponding *in vitro* system almost undetectable [25]. This patient too developed myoclonic epilepsy.

All the evidences suggest that when Asn188Ser is coupled with null mutations, like recombinant alleles, or severe mutations with no enzymatic residual activity, the resulting phenotypes are severe, i.e. neurological [36]. Our report strengthens the association of Asn188Ser with the particular neurological phenotype of type 3 GD patients, expanding the number of reports in which the Asn188Ser is *in trans* with null mutations.

## Conclusions

We stress the importance of considering synonymous variants and thoroughly investigating their possible pathogenicity on natural acceptor and donor sites and, also, on possible rescue or removal of ESE and ESS sequences.

Identifying which nucleotide changes represent benign polymorphisms and which may instead result in potential disease-causing mutations is challenging for diagnosis, especially in cases of synonymous variants. Our data show that the risk for underestimating synonymous mutations can be reduced or altogether avoided by *in silico* predictions and mRNA evaluation/quantization.
